# Incorporation of Graphene-Related Carbon Nanosheets in Membrane Fabrication for Water Treatment: A Review

**DOI:** 10.3390/membranes6040057

**Published:** 2016-12-19

**Authors:** Jenny Lawler

**Affiliations:** School of Biotechnology and DCU Water Institute, Dublin City University, Dublin 9, Ireland; jenny.lawler@dcu.ie

**Keywords:** carbon nanosheets, graphene, graphene oxide, water treatment

## Abstract

The minimization of the trade-off between the flux and the selectivity of membranes is a key area that researchers are continually working to optimise, particularly in the area of fabrication of novel membranes. Flux versus selectivity issues apply in many industrial applications of membranes, for example the unwanted diffusion of methanol in fuel cells, retention of valuable proteins in downstream processing of biopharmaceuticals, rejection of organic matter and micro-organisms in water treatment, or salt permeation in desalination. The incorporation of nanosheets within membrane structures can potentially lead to enhancements in such properties as the antifouling ability, hydrophilicy and permeability of membranes, with concomitant improvements in the flux/selectivity balance. Graphene nanosheets and derivatives such as graphene oxide and reduced graphene oxide have been investigated for this purpose, for example inclusion of nanosheets within the active layer of Reverse Osmosis or Nanofiltration membranes or the blending of nanosheets as fillers within Ultrafiltration membranes. This review summarizes the incorporation of graphene derivatives into polymeric membranes for water treatment with a focus on a number of industrial applications, including desalination and pharmaceutical removal, where enhancement of productivity and reduction in fouling characteristics have been afforded by appropriate incorporation of graphene derived nanosheets during membrane fabrication.

## 1. Introduction

**Figure 1:**
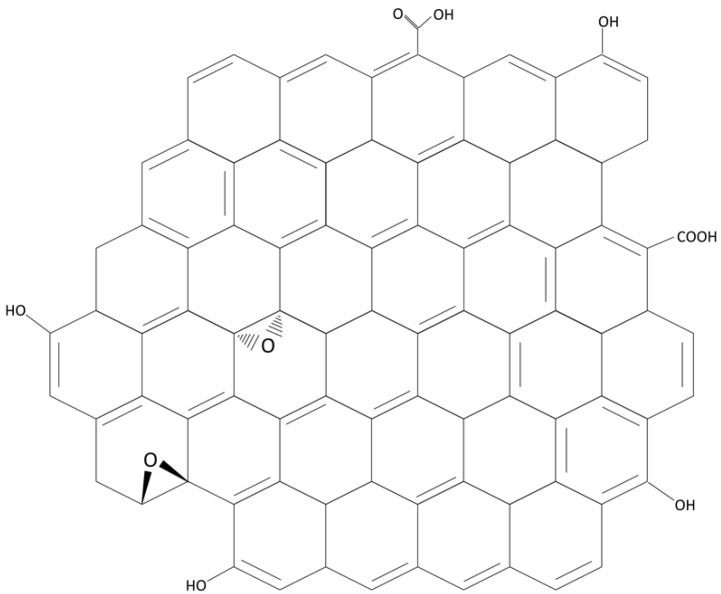
Graphene oxide (GO) structure.

Nanosheets provide the opportunity to produce tailored membranes by allowing manipulation of membrane structural properties, hydrophilicity, charge and charge density, thermal and mechanical stability, and porosity and roughness; they have the potential to enhance the balance between permeability and selectivity while minimising the propensity for fouling that can be the major limitation of membrane separations [1,2]. Graphene is a two-dimensional single-layered nanomaterial arranged in a hexagonal honeycomb lattice structure, consisting of sp^2^-hybridized carbon atoms (Figure 1). Carbon nanosheets such as graphene and derivatives such as graphene oxide (GO) and reduced graphene oxide (rGO) have received increasing attention in the membranes literature due to their interesting properties and potential for enhancing the performance of polymeric or ceramic membranes by fabrication of nanocomposites or nanohybrids. Research ranges from application of pure graphene films and graphene in which nanopores have been introduced, to GO and rGO, in which nanoscale defects naturally occur during the manufacture process. The resulting membranes, a combination of polymers or ceramics and nanomaterials, are increasingly being seen as a solution to, in particular, environmental problems such as the challenge of providing clean water free from contaminants, or clean fuel. Nair et al. [3] showed that thin graphene oxide (GO) membranes allowed transport of water only, but did not allow passage of gases or other liquids, attributed to the low-friction flow of a monolayer of water through capillaries formed by closely spaced graphene sheets. GO has an ultra-thin two dimensional structure and has been shown to possess many oxygen containing functional groups (e.g., carboxylic acid groups at edges, and phenol hydroxyl and epoxide groups at the basal planes), which can confer hydrophilic properties on the membrane [4], and provide reactive sites for membrane functionalization, thus displaying excellent water permeability (Figure 1). Water flow is afforded due to the spacing between nanosheets, which is typically of the order of 0.3–0.7 nm, which is ideal for the permeation of water while blocking the transport of larger molecules [3]. Spacing between nanosheets leaves interconnected channels through which water molecules can flow, and a convoluted tortuous path is generally accepted to follow mainly over the hydrophobic non-oxidized surfaces rather than the oxidized, hydrophilic regions [5,6]. Mass transport is generally governed by steric exclusion, electrostatic interaction, and chemical binding in the case of GO and rGO [7].

## 2. Strategies for Membrane Fabrication

**Figure 2:**
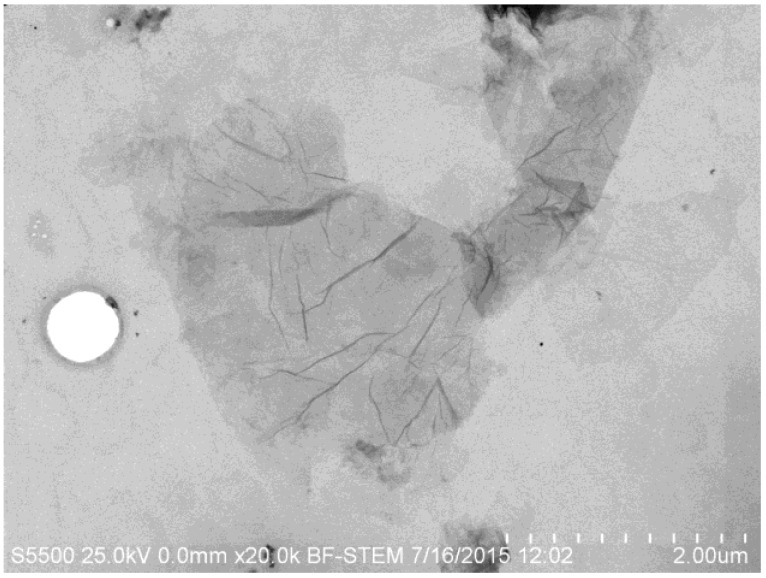
FESEM Image of GO in Transmission mode.

**Figure 3:**
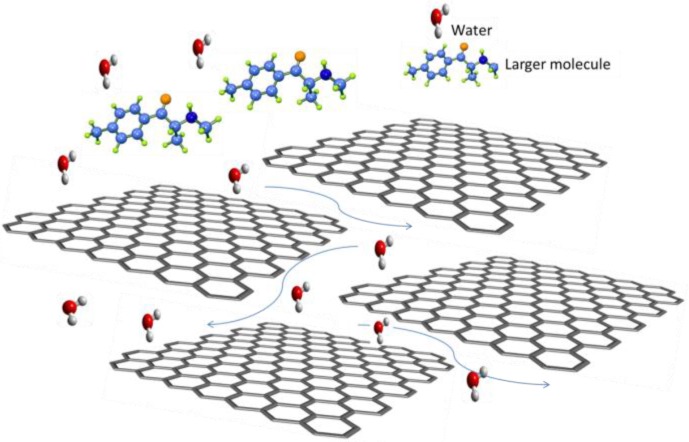
Transport in GO stacked layers.

Graphene derivatives such as GO are attractive due to their ease and low cost of manufacture in comparison to pristine graphene. GO is typically produced from an aqueous dispersion of graphite oxide, followed by exfoliation in a solvent, typically using a Hummer’s or modified Hummer’s and Offeman method [8,9]. Figure 2 shows clearly the thinness of the GO nanosheet, while the wrinkled surface is clearly visible. The pore size/nanosheet spacing is adjustable, by tuning the properties of the GO nanosheets including by inclusion of filler molecules such as TiO_2_ or other nanoparticles [5,10,11,12]. Transport of molecules larger than the void spacing of the stacked GO sheets is blocked, whereas permeation of water is extremely fast (Figure 3) [5,13].

Fabrication of many nanocomposite membranes is based on phase inversion (e.g., the non-solvent induced phase separation (NIPS) method or thermally induced phase separation (TIPS) method), in which nanosheets are dispersed in polymer solutions prior to the casting of porous or microporous membranes, and can be prepared in either flat sheet or hollow fiber configurations. During the NIPS process, the hydrophilic nature of GO sheets means that the GO converges to the membrane and pore surfaces [14] during precipitation in aqueous solution. This type of membrane is mainly used in MF or UF processes due to its typically porous structure, and the structure most often consists of a thin dense skin layer over a more macroporous sublayer (asymmetric structure), formed due to the slower precipitation of the polymer below the skin layer [7]. More dense membranes, such as NF or RO membranes, are typically of a thin film composite (TFC), multi-layer type structure, composed of a thin dense polymer film or barrier layer on top of one or more porous support layers [15]. The porous membranes previously outlined often form the porous support layer for TFC membranes, and carbon nanosheets can be incorporated into either the support layer or the thin barrier layer. GO membranes have been prepared by layer-by-layer (LbL) deposition of GO suspension or else simple vacuum filtration onto a support layer [16], while membranes have also been prepared by electrospinning, spin coating or drop casting [17,18]. While vacuum/solution filtration for deposition of pure GO membrane layers appears attractive, the mechanical stability under hydrated conditions can be insufficient, with GO layers tending to slough off the support layer under typical crossflow/pressurized conditions. In an aqueous environment, the layers become negatively charged, leading to electrostatic repulsion that can exceed the van der Waals or hydrogen bonding forces that hold the GO layers together [11,19]. As such, surface layer deposited GO must generally be crosslinked to maintain membrane integrity [16,20], and this is often an approach taken for immobilization of GO on the surface of ceramic membranes.

## 3. Carbon Nanosheet Membranes for Desalination

Fresh water scarcity is an increasing problem globally, which has led to a strong drive for research in the area of water reclamation and seawater desalination. The need for fresh water both domestically and industrially (agriculture, food production etc.) has increased at unprecedented rates in recent years. The most common type of membrane for water desalination is reverse osmosis (RO), as it rejects mono- and divalent salts from sea and brackish water, while energy requirements for RO have decreased to levels that are more desirable than thermal methods [21]. Reverse osmosis utilises two opposing forces in effecting separation between two phases, the concentration gradient and the pressure gradient. During osmosis, flow of pure water through the membrane to the salt rich side will occur. During reverse osmosis, high pressure is applied on the salt rich side of the membrane, counteracting the osmosis effect of the concentration gradient.

In recent years, alternative membrane processes such as forward osmosis (FO) and nanofiltration (NF) have garnered increasing attention in the literature and industrially, as they have some potential advantages including lower energy requirements.

Nanoporous graphene has proven to be an excellent candidate for desalination, with membranes consisting of nanopores created in single layer graphene sheets using an oxygen plasma etching process displaying almost 100% salt rejection with extremely fast fluxes of 6 × 10^5^ L/m^2^/hr (LMH) [22], and similar membranes prepared using double layer graphene sheets etched using selective ion beam etching also showing great promise [23], under pressure driven conditions.

While graphene itself would be an excellent candidate for removal of salts while offering fast water transport, due to difficulties in large scale manufacture and stability as previously discussed, GO or rGO are the most attractive options currently for membrane preparation/modification.

The most commonly employed membranes for desalination have been polyamide thin film composite (PA-TFC) membranes, due to their good mechanical stability and excellent separation capabilities. PA-TFC membranes typically consist of a non-woven fabric support layer of approx. 120 µm thickness, topped with a sublayer of polymer (often polyethersulfone (PES) or polysulfone (PSf) with a thickness of approximately 40 µm, on which an interfacial polymerization is performed to fabricate a surface active polyamide layer of approximately 200 nm. The surface active PA layer is typically the part of the membrane that governs the separation process. This type of membrane has superseded the previously employed cellulose acetate (CA) membranes, as they can tolerate larger ranges of operating pH, temperature and pressure. Although improving, energy consumption and membrane fouling remain a problem in RO membranes, and this is an area of much research. Fouling mitigation measures such as enhancing the hydrophilicity and decreasing the surface roughness of the membrane is a common approach for improving membrane performance. Chlorine resistance is another pressing issue—chlorine residues that typically remain in pre-treated water can be damaging to the membrane surface active layer. Mitigation of biofouling via the membrane itself is something that has potential—with incorporation of antiomicrobial compounds such as copper or silver in hybrid nanocomposites with GO or rGO showing synergistic effects in water disinfection [24,25].

Modification and functionalization of both surface active layers and membrane sublayers have been investigated, and the incorporation of nanostructures such as GO shows great promise due to fast water channels, high permeability and excellent hydrophilicity [26,27]. It is possible to modify the membrane properties either by surface modification (such as coating or grafting) or by modification of the composition of the sublayer or active layer, for example using amide coupling between carboxyl groups of graphene oxide and carboxyl groups of the polyamide active layer [28]; in addition the mechanical properties of the membrane can be enhanced for example by crosslinking with materials such as chitosan [29].

LbL deposition of GO multilayers coated on a PA-TFC RO membrane displayed an increase in surface hydrophilicity and decrease in surface roughness, leading to improved antifouling characteristics. The GO layer acted as a protection against chlorine damage to the PA layer, so cleaning or chlorine exposure did not result in degradation of salt rejection [30]. The LbL deposition of negatively charged graphene oxide (GO) nanosheets on a porous poly(acrylonitrile) support yielded a tight nanoporous membrane with molecular weight cut-off (MWCO) of <350 Da, ideal for multivalent ion desalination/water softening in FO process [13]. 0.02 wt % rGO-TiO_2_ nanocomposite embedded in the PA layer of TFC RO membranes yielded significant improvements in chlorine resistance, antifouling ability, hydrophilicity and enhanced surface roughness, with fluxes of >51 LMH and good salt rejection [20], while GO-TFC membranes produced by incorporation of GO in the PA layer yielded poorer salt rejection (~93% NaCl rejection) but better flux (59 LMH) [4]. Membranes prepared via GO-monolayer titania with UV reduction from GO to rGO demonstrated excellent ion rejection (<5% permeation) with good water permeabilities provided by photoinduced hydrophilic conversion of the titania [31]. Incorporation of GO into a PSf support layer with subsequent interfacial polymerization to form PA barrier layer yielded membranes suitable for operation in either RO mode (with salt rejection of almost 99% and flux of almost 2 LMH per bar applied transmembrane pressure), or FO mode [32].

NF membranes sit somewhere in between UF and RO, with “tight” NF membranes being compared to “loose” RO membranes, and “loose” NF membranes being akin to “tight” UF membranes. Looser NF membranes are of significant interest for water softening, for example with layer-by-layer assembled polyethyleneimine-GO membranes exhibiting a positively charged surface for salt rejection [33]. Tight NF membranes have a salt rejection for Na^+^ of ~90%, and typically have a MWCO of <200 Da. Many parameters of the membrane are implicated in the control of solute rejection and flux, including the operating conditions, membrane fabrication technique, composition of the active layer (and sublayer), and presence and concentration of active layer cross-linking agent. Transport of solutes across the membrane is similar to RO membranes in terms of diffusional limitation and steric exclusion. The solute rejection depends strongly on the solute type, which includes charge valency, diffusion coefficient, and hydration energy [34]. The flux in NF membranes tends to decrease with increasing salt concentration e.g., NaCl or Na_2_SO_4_ in the feed solution, due to the increase in feed osmotic pressure [35] or decrease in GO zeta potential with increase in NaCl concentration in lamellar GO membranes [36]; while decrease in surface roughness tends to improve filtration characteristics by increasing the antifouling properties of the membrane. Solute rejection was shown to be improved when chemical functional groups (such as sulfonic or carboxylic acid groups) were incorporated into NF membranes [37], while allowing for a decrease in pressure requirements and allowing for thinner membranes with a concomitant increase in permeate flux, while in addition, an increase in hydrophilicity of membranes was shown to enhance their antifouling capabilities [38], suggesting that GO would be an excellent target for research into NF membrane modification. In addition, molecular dynamic simulations have shown that graphene layers with nanopore diameters could offer high salt rejection and excellent fluxes, far improving on what is currently possible with RO membranes [39,40,41], supported by recent experimental work [22,23].

**Figure 4:**
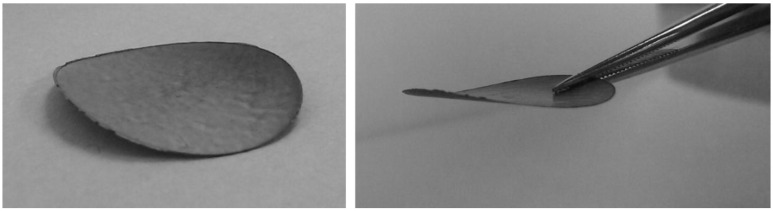
Freestanding reduced graphene oxide (rGO) films.

Vacuum deposition of GO based films onto support membranes has been used to prepare tight NF membranes (Figure 4). Ultrathin (<60 nm thick) rGO layer on polymeric MF membranes provided good salt rejection (20%–60% of ions) [42], while better salt rejection but lower flux were found for membranes with MWCNTs incorporated into the rGO nanolayer when deposited on Al_2_O_3_ MF membranes [43]. The antifouling nature of the membranes to HA and sodium alginate was good but protein fouling was not ideal. Improvements in flux due to enhancement in steric effects were realized by better dispersion and mixing of the CNT/GO layer by addition of pluronic surfactants (block copolymers) which were vacuum filtration deposited onto anodic Al_2_O_3_ MF membranes [44]. Again, drying related shrinkage of films of this type have been reported and damage to films is possible under crossflow and high TMP conditions, although efforts to stabilise the film showed reasonable success by incorporation of a sacrificial layer of PMMA with in-situ reduction of GO using UV [45]. Dip coating of negatively charged GO film onto positively charged polyamide-imide/polyethylene-imine hollow fibre membranes yielded a significant flux enhancement without sacrifice of salt rejection, with good mechanical stability of dip coated layer [46].

Interfacial polymerization of GO/rGO/polymer mixtures on support layers has also been shown to be effective in preparation of NF membranes. Excellent salt rejection of 88% for NaCl and 97% for MgSO_4_, with the latter enhanced by electrostatic repulsion due to the membrane charge, were achieved using membranes prepared by interfacial polymerization of mixtures of rGO and polyamide (PA) on PSf UF membrane supports [47]. The addition of rGO was shown to enhance the membrane antifouling properties over pure PA membranes when tested with HA and BSA. rGO-TiO_2_ has been included as a hydrophilic modifier in the active layer of TFC membranes, leading to a decrease in surface roughness and increase in the fouling resistance of the membranes. Fluxes of ~55–60 LMH were achieved, with >90% rejection of the multivalent SO_4_^2−^ anion and 35% of monovalent chloride anion [20]. Increase in the rGO-TiO_2_ content of the membrane above 0.02 wt % led to a decrease in the salt rejection, potentially due to increase in defects in the active layer. Ring opening polymerization reaction was used to stably immobilize epoxy groups of GO to amino groups of an active layer of *O*-(carboxymethyl)-chitosan on a PSf membrane support. The GO nanosheets were immobilized on the surface, and again increase in negative charge was seen with rejections of >97% for Na_2_SO_4_ and >60% for NaCl, with fluxes of >20 LMH [48]. Loose NF membranes with zwitterionic GO grafted with poly(sulfobetaine methacrylate) brushes (GO-PSBMA) were prepared by phase inversion of mixtures of GO-PSBMA and PES [49]. The zwitterionic nature of the membranes allowed for reduction in fouling potential, and the loose nature of the membranes allowed retention of dyes but permeation of salts [50]. Polyethylene glycol modified graphene oxide (PEG/GO) nanosheets were incorporated into the PA layer, with UV irradiation shown to enhance permeate flux while maintaining rejection of NaCl and Na_2_SO_4_ of 63.5% and 93% respectively [28]. The flux enhancement was attributed to the standard increase in hydrophilicity due to inclusion of GO in the structure, with UV irradiation leading to longer hydrophilic PEG backbone in the structure of the thin layer.

While reverse osmosis is the most commonly used membrane based desalination method, and NF is gaining increased interest particularly for water softening, pervaporation (PV) has attracted attention as an alternative approach in recent years as it offers lower energy consumption and ease of operation, not requiring the high pressures of RO/NF separation [16]. Pervaporation utilises a dense membrane (polymeric, inorganic or hybrid) through which a specific component may preferentially permeate and evaporate. The feed is typically heated to approx. 45–60 °C, allowing the use of low-grade waste or solar energy. A gradient of concentration and vapour pressure drives the mass transfer. A low vapour pressure on the permeate side is generated by either vacuum, an air gap, or a sweep flow, and it is generally accepted that the separation is effected under solution-diffusion mechanism [51]. While pervaporation has mostly been utilized for solvent dehydration and separation of organic mixtures, it demonstrates high salt rejection (above 99%) due to the non-volatile nature of the salts. The impact of feed concentration on permeability is less in PV than in RO, as the high osmotic pressures that have to be overcome in RO do not apply in PV due to the phase change nature of the mass transfer (rather than pressure driven)—this means that PV is well suited to handle high salinity feeds, and can be more fouling resistant than RO. PV membranes typically do not suffer with fouling from adsorption or pore plugging, and membrane wetting and salt leakage can be avoided.

The flux in PV membranes remains low, typically less than 6–10 LMH, and is strongly dependent on the physicochemical characteristics of the membrane material. As such significant interest lies in the exploration of highly hydrophilic materials (such as GO) with open passages for water transport, for high flux while maintaining salt rejection.

Membranes utilizing a 3-dimensional GO framework supported on Al_2_O_3_ tubular membranes by covalent synthesis and vacuum filtration have been prepared, using crosslinking agents to prepare the 3-D framework, allowing separation of the GO layers, enhancing mechanical stability while allowing flux and rejection of salts. Salt rejection of >99.5% has been achieved with fluxes >11 LMH using 1,4-phenylene diisocyanate (PDI) crosslinker [16] and >48 LMH using PDA [52].

In a process similar to pervaporation, membranes suitable for direct contact membrane distillation were prepared by immobilization of GO on the surface of a hydrophobic polytetrafluoroethylene (PTFE) membrane, achieving fluxes of up to 97 LMH [53].

## 4. Photocatalytic Carbon Nanosheet Membranes

In order to address the issue of membrane fouling discussed previously, efforts have been made to incorporate photocatalytic elements into membranes as foulant degradation can be carried out in-situ, hence reducing the requirement for chemical cleaning or membrane replacement. Photocatalyst compounds can be incorporated within the membrane matrix or deposited or bonded onto the membrane surface, depending on the application.

A graphitic carbon nitride nanosheet/reduced graphene oxide/cellulose acetate composite photocatalytic membrane (g-C_3_N_4_ NS/rGO/CA) was prepared by vacuum filtration onto the surface of a commercially available cellulose acetate (CA) microfiltration membrane, with increase in flux over CA membrane alone. While the inclusion of rGO within the membrane matrix enhanced the antifouling properties due to increase in hydrophilicity, visible light irradiation further enhanced the flux owing to degradation of organic compounds responsible for membrane fouling [34]. Titanium dioxide (TiO_2_) is one of the most common photocatalysts employed, and GO has been shown to enhance the photocatalytic activity of immobilized TiO_2_ as GO sheets can facilitate separation of photo-induced electron-hole, suppressing charge recombination [54]. GO-TiO_2_ can be prepared via simple mixing or via sol-gel process, to avoid agglomeration of TiO_2_ nanoparticles at higher concentrations [12].

GO/TiO_2_/Fe_2_O_3_ was deposited via vacuum filtration onto glass fibre filters, exhibiting enhanced adsorption of HA with photocatalytic degradation under simulated solar irradiation under constant flux of 145 LMH, with 40% of the pressure drop associated with the same membrane under dark conditions [55]. GO-TiO_2_ photocatalytic membranes have been prepared by LbL deposition onto PSf support [56], as a microspherical hierarchy onto CA membrane [57], immobilized on alginate hollow fibres via a wet-dry spinning process [58], decorated onto poly(vinylidene difluoride-*co*-trifluoroethylene) (P(VDF-TrFE)) copolymer fibres and electrospun to prepare nanofibrous mats [59] and immobilized by dip-coating onto ceramic UF and NF monoliths [60]. In all cases increase in membrane flux due to enhanced hydrophilicity and degradation of pollutant under both UV and visible light was observed.

## 5. Nanofiltration for Pharmaceutical Removal from Water

The release of wastewater treatment plant (WWTP) effluent can be considered a major source of pollution in drinking water sources, along with contamination from landfill leachate, sewage system or septic tank leakage, and contamination from agricultural runoff, as surface and groundwaters account for a significant proportion of drinking water sources. One group of compounds that has attracted increasing interest in recent years is that of so called “contaminants of emerging concern”, such as pharmaceuticals and personal care products (PPCPs) or hazardous organic pollutants (HOPs). Concentration of PPCPs in drinking water sources will be typically at least an order of magnitude less than that observed in WWTP effluents, due to the processes of dilution and sorption to solids, but degradation of these compounds is unlikely [61].

Many PPCPs have been implicated as having endocrine disrupting properties, affecting hormone levels and reproductive function of aquatic animals, promoting the increase in antibiotic resistance of microorganisms, and other both chronic and acute health effects. While levels found in drinking water sources are typically very low (of the ng/L to μg/L order of magnitude), the effects of long term exposure to low levels of these chemicals is unknown [62,63]. In addition, certain compounds such as Diclofenac have been found in mg/L levels [64]. Many of the transformation products or metabolites of these substances can themselves be considered contaminants of concern [65]. A number of studies on the occurrence of PPCPs in various matrices have shown that a wide variety of pharmaceuticals at varying concentrations occur in WWTP effluents [66], WWTP influent and effluent streams [67,68], in sewage sludge and in sludge enriched soils [69] and in the marine and wider aquatic matrices [70,71]. In some cases (e.g., carbamazepine) the effluent concentrations from WWTPs was found to be higher than the influent concentration [67,72]. WWTP effluents containing PPCPs are a good indication that the local drinking water sources may be contaminated. Many drinking water treatment plants do not have the technical capacity to remove micropollutants such as pharmaceuticals and other trace organic compounds [73]. Although adverse health effects from long term, low level exposure to these compounds is not yet proven, drinking water treatment facilities should be designed to provide water that is free from pharmaceuticals [74].

Regulation standards for drinking water (including that from reclaimed water) are designed to ensure that the water is of a reliable and safe quality. However, discharge guidelines from wastewater treatment plants (WWTPs) for many PPCPs are not yet encompassed in the common regulatory frameworks, likewise there remains a lack of regulation for limits in drinking water. The European Water Framework Directive (WFD) [75] contains a dynamic list of priority pollutants which should be regulated and monitored in WWTP effluents [66]. 12 new substances have recently been added to the original 33 priority pollutants and 8 other pollutants, with a new requirement for monitoring and reporting of 3 pharmaceutical compounds (an anti-inflammatory drug, diclofenac, and two hormones, 17α-ethinylestradiol and 17β-estradiol) [70]. These compounds now appear on the EPA Watch List, with the potential for future addition to the list of priority pollutants. In order to assure water quality and future proof the potential for reclaimed water use for potable purposes, the development and application of treatment technologies that are capable of removing PPCPs is critical.

Typical drinking water treatment plants consist of a number of stages, including chemical coagulation/settling, sand filtration, adsorption using granular activated carbon (GAC) and disinfection using chlorination, ozone, UV or a combination of these technologies. Chemical coagulation has shown limited potential for removal of pharmaceuticals, reducing the concentrations of ionisable pharmaceuticals only but not removing them completely, while non-ionisable pharmaceuticals such as carbamazepine were unaffected [72,76]. Sand filtration has been shown to be ineffective in the removal of pharmaceuticals such as bezafibrate, clofibric acid, carbamazepine and diclofenac, while high dose ozone (3 mg/L) was not capable of removing clofibric acid [77]. UV was shown to be ineffective in the removal of antibiotics, although chlorination and ozone were able to reduce concentrations to a reasonable level [78]. Residual free chlorine in the drinking water treatment system was shown to be capable of further degradation of approximately 50% of a cohort of 98 studied pharmaceuticals in treated drinking water [79]. However, due to the nature of many pharmaceuticals (polar, with acidic or basic functional groups), ozonation or chlorination can result in transformation of compounds rather than removal [80]. Thus there is a driver for physical removal rather than transformation, a function which membrane separation processes such as nanofiltration could reasonably perform. There is very little research on the application of graphene enhanced nanofiltration membranes on the removal of PPCPs from drinking or wastewater streams, and given the promising results in other areas such as desalination, this is an area that is worthy of increased attention. The background to the issue and the limited research in the area is presented in this section.

While reverse osmosis (RO) is an established unit operation in the advanced treatment of water, the application of nanofiltration (NF) is a membrane separation process that is gaining increased attention in the literature for the removal of trace organic contaminants from drinking water. Nanofiltration is a medium-to-high pressure (7–40 bar) crossflow membrane filtration process employing membranes with pore sizes in the range 0.5–5 nm and MWCO in the range 200–2000 Da. It is has been shown to remove protozoa (including oocytes), bacteria and viruses, as well as natural organic matter (NOM), organohalides, pesticides and pharmaceuticals [81,82]. NF compares favourably with RO, in that it can be operated at lower applied pressure and rejects fewer mineral ions—meaning that the energy requirements are less, and that the permeate needs less post-treatment [74,83].

While it would appear at first glance that most pharmaceuticals, which have molecular weights within 150–500 Da, should be automatically rejected by NF membranes, NF does not operate simply on a size exclusion basis. Many characteristics of both the pharmaceuticals and the membranes themselves can affect solute rejection, including their polar/non-polar nature, acid disassociation constant, hydrophobicity/hydrophilicity, molecular weight and molecular size, and membrane charge. Typically the only information supplied by NF membrane manufacturers is the MWCO, and the molecular weight of a component which will have a 90% rejection, implying that larger molecules will have a rejection of more than 90%, while smaller molecules will have a rejection of less than 90% [84]. This, however, is often not the case, for example where size exclusion may dominate the rejection in one membrane type, rejection for another membrane material even with similar pore size may be dominated by electrostatic interactions [85], and there is a large variation in the literature between reported rejection/retention values for pharmaceuticals [86].

Rejection or retention of pharmaceuticals on NF membranes is governed by the operating parameters (e.g., crossflow velocity, operating temperature, transmembrane pressure), solute properties (e.g., charge, hydrophilic/hydrophobic, polar/non-polar), membrane properties (e.g., surface roughness, charge, pore size/MWCO, functional groups (acidic/basic) and feed composition (e.g., pH, ionic strength, presence of other substances such as humic acids)) [74,87,88]. The most well understood mechanism of rejection of solutes by NF is the physical sieving of solutes larger than the MWCO of the membrane, while steric effects will also lead to rejection. Other physico-chemical interactions such as adsorption and electrostatic diffusion are also important in rejection, as are charge exclusion effects (electrical and Donnan). Hydrophobic-hydrophobic interactions between the membrane and solute and diffusion limitation of the solute is also seen to play a role [37,89,90]. Steric hindrance is the most likely mechanism for rejection of uncharged, hydrophilic compounds, and correlation between the molecular size/weight and the membrane pore size distribution has been seen in the rejection of solutes of this type [84,91].

Molecules that are cylindrical in shape can display a tendency to approach the membrane pores in a vertical manner, due to the polarity of the molecules (dipole moment) being attracted to fixed charge groups on the membrane surface. Thus low retention of high dipole moment organic compounds can be influenced by steric effects [92]. Molecules with low dipole moment such as diclofenac and ibuprofen have been shown to have greater rejection than molecules such as carbamazepine [93]. Less polar (low dipole moment) molecules can be preferentially adsorbed to the membrane, particularly if they are also hydrophobic. Adsorption typically results in a lower steady state rejection, and a time dependent effect in NF will be observed as the adsorption capacity of the membrane is reached [73]. Removal efficiency of solutes is also related to their octanol water partition coefficient (log K_OW_), which is related to their hydrophobicity, and interaction with hydrophobic membranes. High log K_OW_ (compounds such as diazepam, ibuprofen and diclofenac) and low solubility can lead to an increase in rejection, and this in turn can in part be attributed to hydrophobic adsorption to the membrane surface [94,95]. Ibuprofen, for example, in its neutral form, has a relatively high hydrophobicity, and reasonably high levels of adsorption to hydrophobic membrane surfaces will typically be observed. In the presence of other solutes which also adsorb to the membrane surface (competitive adsorption), it may be observed that an increase in rejection occurs for compounds that sorb less in comparison with other compounds within a mixed matrix, in comparison to the rejection that would be observed in a single-component feed [96,97]. In addition, adsorption can lead to a decrease in the time required to achieve steady state rejection, leading to overestimation of the short-term rejection capabilities of membranes even for compounds with a low log K_OW_ [98,99].

**Figure 5:**
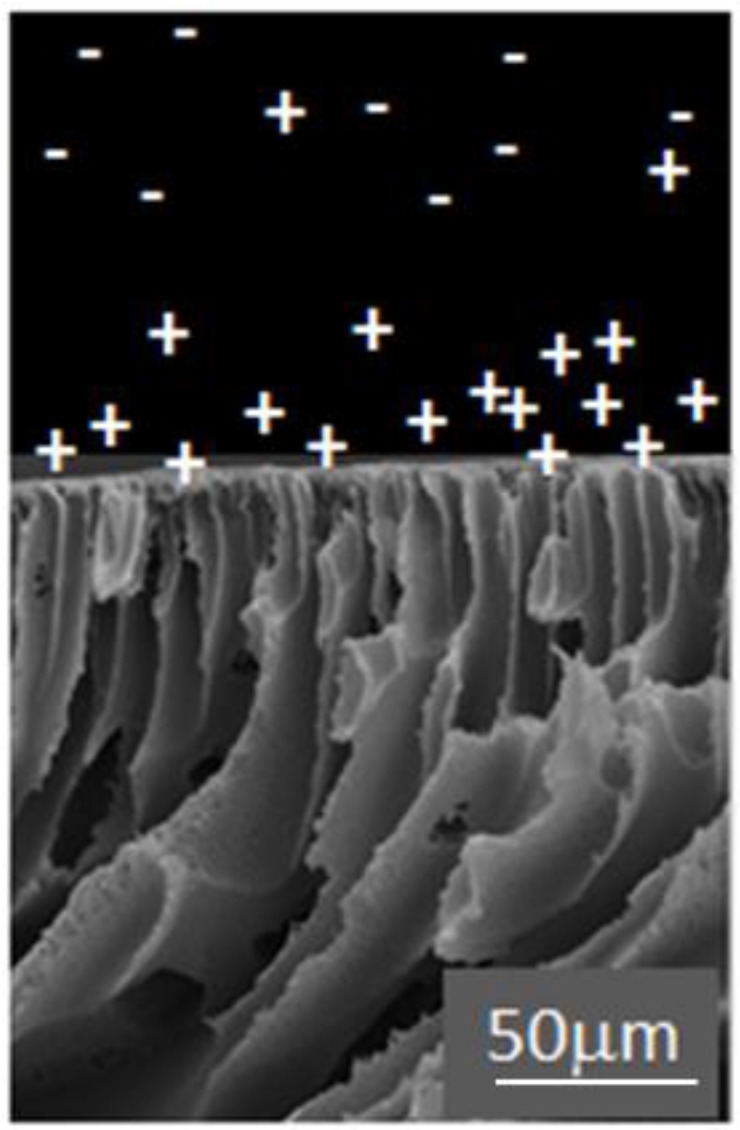
Charge concentration effect—visualization with SEM image of membrane with GO-TiO_2_ (1 wt %)/polyethersulfone (PES) porous support layer.

Electrostatic repulsion is seen to play a role, where rejection of solutes with the same charge as the membrane is enhanced [37,100]. For negatively charged membranes, negatively charged solutes are better removed compared to uncharged solutes, while positively charged solutes demonstrate poor removal. However membrane shielding effects can be observed for solutes with the opposite charge to the membrane with increasing solute concentrations, due to charge shielding effects on the membrane surface [101,102]. A “charge concentration effect” may be observed, where positively charged and neutral molecules engage in hydrophobic interactions with a negatively charged membrane, while negatively charged molecules cannot approach the membrane surface (Figure 5). Thus increased concentration of positively charged solutes will be seen at the membrane surface in comparison with the bulk fluid [91,103].

The pH and p*K*_a_ values of the solutes have a significant effect on the retention of PPCPs on charged membranes, particularly for ionizable pharmaceuticals such as sulfamethoxazole and ibuprofen, whereas it is less important for non-ionizable compounds such as carbamazepine. Retention on negatively charged membranes can be seen to increase as the compound becomes negatively charged above its p*K*_a_ value, for example the rejection of amoxicillin was enhanced by 85% with increase in pH above the p*K*_a_ [104]. Rejection of ibuprofen (hydrophobic and acidic) at pHs lower than the p*K*_a_ led to reduction in diffusion through the membrane due to partial adsorption on a negatively charged membrane surface, while electrostatic repulsion was dominant at higher pH [105]. High ionic strengths can minimise the contribution of electrostatic interactions on rejection, by suppression of the double layer or Debye screening length, although this effect is thought to be not as significant, due to the formation of a hydrated layer around the charged functional groups of the pharmaceuticals [106]. The speciation of pharmaceuticals (i.e., the different forms in which they occur, and the transition between those forms) can also lead to change in retention as a function of pH [92]. For example, Clonazepam is protonated at highly acidic conditions and it becomes non-protonated (neutral) when the pH is increased up to 6. However, under alkaline conditions, the compound changes to its enolic form, which has an enhanced water affinity due to the charge on the molecule; this in turn leads to reduction in retention [94].

The mechanism of transport through NF membranes is generally thought to be a combination of solution/diffusion processes, as well as flow through defects or pores in the nanometer scale on the membrane surface [96,107,108]. The concentration difference across the membrane is a driver for flux of solutes through the membrane by diffusion. It has been shown that an increase in water recovery, increasing the concentration differential across the membrane, leads to a decrease in rejection of solutes [109]. The solute and water transport through the membrane pores can be enhanced by increasing the cross-flow velocity or transmembrane pressure, and it has also been shown experimentally that this can lead to an increase in rejection due to a reduction in adsorption effects [110].

The ability to predict the performance of NF in rejection of PPCPs has been a subject of increasing research. Mathematical models have been developed that incorporate various combinations of feed, operating, membrane and solute parameters in an effort to develop robust predictions. Early attempts at modelling rejection in NF systems were based on either variations of Hermia models or Nernst-Planck equations for mass transfer. In modified Hermia models, fouling/rejection in larger pore size membranes was governed by both size exclusion and electrostatic effects while in smaller pore size membranes size or steric effects were assumed to dominate, and good agreement with prediction was found for the rejection of acetaminophen and sulfamethoxazole [111]. Models based on the extended Nernst-Planck equation for mass transfer were developed, taking the molecular size of solutes and hence hindered transport through narrow pores into account. This model accounted for convection, ionic diffusion and electromigration of species within the membrane pores. Donnan equilibrium was assumed to be the electrostatic interaction involved in ion partitioning between the membrane and liquid phases [112,113]. Other research has focussed on the development of membrane transport models, to elucidate the contributions of diffusion and convection on solute transport and rejection. Modelling studies have shown that diffusion is important for hydrophobic non-polar compounds while convection dominates for other molecules, and that convection is more important for loose NF membranes [89]. Analytical models for solute transport and rejection have also been developed, such as those incorporating the free energy of interaction and the size exclusion contribution, with good agreement between prediction and experiment achieved for a range of hydrophilic/hydrophobic/charged/uncharged pharmaceuticals was achieved by Botton et al. [38].

This type of model was further developed by inclusion of the dielectric potential as part of the ion partitioning effect, termed the Donnan Steric Pore Model and Dielectric Effect (DSPM&DE) [114]. This model was deemed quite simple to use, involving just three adjustable parameters: the surface charge density, average pore size, and effective membrane thickness, and was applied in prediction of rejection of hormones and some PPCPs. The inclusion of dielectric effect enhanced the realistic behaviour predicted by the model [115]. However the rejection was often overestimated, due to the underestimation of the contribution of hydrophobic interactions with the membrane [92]. The model was refined to take this affinity effect into account, and predictions were improved for hydrophilic compounds including acyclovir, caffeine and ranitidine, however retention of hydrophobic molecules was still over-estimated [74], despite extensive model verification for salts [116]. This model was incorporated into a freely available computer programme, NanoFiltran, developed by Geraldes and Brites Alves [117] and further developed by incorporation of the increase in solute concentration along the membrane module for seawater desalination [118], however it has not been applied to PPCPs in the literature. While the DSPM&DE type models are suited for charged solutes, models based on the Spiegler-Kedem-Katchalsky equations (SKK model) has been successfully applied to neutral molecules, including the pharmaceuticals isopropylantipyrine and antipyrine [119]. A quasi-empirical model combining predictions for neutral and charged solutes (taking pH dependency into account) was applied reasonably successfully for carpamazepine, ibuprofen, sufadiazine, sulfamethoxazone and sulfamethazone [50]. This simplified charge concentration polarization (SCCP) model can predict the effects of electrostatic interactions on the rejection of trace organic solutes [103], however without the presence of charge interactions (e.g., at pH lower than p*K*_a_), experimental measurements must be incorporated into the model.

The effect of organic fouling on membrane characteristics is typically found to be membrane dependent, with pore size deemed to be the dominant factor. For example, fouled tight NF membranes have been shown to become more hydrophilic and negatively charged, whereas loose NF membranes became more hydrophobic and less negatively charged. The membrane physical characteristics also play a role, with hydrophobic membranes with high surface roughness being subject to increased levels of adsorption of organics over smooth, hydrophilic membranes [111,120,121]. The major compounds in drinking water sources that have the tendency to foul NF membranes include humic acids and polysaccharides, and model foulants to mimic typical organic fractions and colloidal materials in treated secondary wastewater and surface water are often approximated in laboratory studies using a combination of humic acids, bovine serum albumin, alginate and colloidal silica [122,123]. A fouling layer can build up on the surface of the membrane and within the pores, and this can modify the membrane properties such as hydrophobicity and surface charge, and can also lead to cake enhanced concentration polarization [124]. Membrane fouling and its effect on retention of PPCPs is complex, and both enhancement and decreases in rejections have been reported in the literature. Fouling can depend on the characteristics of the membrane and on the characteristics of the dissolved matter in the feed, and synergistic effects may be observed in mixed foulant systems. Membrane properties such as surface roughness, pore size, and hydrophobicity can have an impact on the level of fouling and thus the level of flux decline that would result from this [74]. In addition to fouling by feed components, the solutes themselves can have an impact on fouling, for example flux decline has been attributed to adsorption of compounds such as diclofenac within the pores of loose NF membranes [93]. Fouling of loose NF membranes, where the mechanism of retention may be governed by pore restriction or blocking during fouled conditions, may lead to an increase in retention, while tight NF membranes may experience a cake enhanced concentration polarization effect, due to modification of the surface charge. Foulant deposition is dependent on feed characteristics, for example with calcium carbonate and sulfate depositing in the early stages of filtration and organic foulants gradually forming a dense packing layer subsequently [125]. Increased hydraulic resistance due to a mixed cake layer structure can enhance flux decline, with adsorption of organics playing a key role, and synergistic fouling can be observed, with more than single layer fouling leading to hindrance of back transport of foulants from the fouling layer [126]. Dissolved organic matter with a medium to low M_W_ can result in membrane fouling by pore blocking, and those with strong hydrophobic characteristics can form a fouling layer, altering the hydrophobicity and surface charge of the membrane [127]. Increased retention of hydrophobic trace organics due to formation of a fouling layer reducing hydrophobic interactions between the solutes and the membrane surface may be observed, with a decrease in diffusional transport across the membrane [128], however, with the decrease in electrostatic interaction based rejection, and change in the surface free energy between membrane and foulants, cake enhanced concentration polarization can lead to decreased rejection [127], where the higher concentration next to the membrane increases the mass transport by diffusion. This has been observed for hydrophilic compounds such as sulfamethoxazole, ibuprofen and carbamazepine with organic fouling on loose NF membranes. The presence of calcium ions leads to increased flux decline, however the cake layers formed can be quite cross-linked and porous, with low hydraulic resistance when alginate or humic acids were the basis of the fouling layers [122]. Calcium ions and humic acid fouling can positively influence the retention of hydrophilic and neutral compounds, where size exclusion is dominant, however concentration polarization decreases the rejection of larger molecules [111]. Biofouling, where microbial layers can build up on surfaces, is another type of membrane fouling that can impact on flux and rejection of PPCPs [38].

Membrane scaling can often occur, particularly in water recycling applications. Scale forming inorganic salts such as CaSO_4_ and CaCO_3_ can interact with organic matter, and have an effect on the flux and rejection characteristics. Humic acids and other natural organic matter can act as anti-scalants, and may act to prevent the expected decline in flux, by forming a porous cake layer. The membrane scaling onset may be delayed by organic matter, leading to a delay in the decline in rejection of PPCPs; however the decline in rejection once scaling begins can be very severe, due to the combined cake-enhanced concentration polarization as a result of the combined membrane fouling and scaling, with the scaling layer leading to an additional concentration polarization effect and reducing shear effects within the underlying fouling layer [86,129,130,131].

Improved rejection of pharmaceuticals can be achieved by tailored fabrication of membranes or modification of commercially available NF membranes. Enhanced rejection by modification of support layer by polymer blending has been investigated [104], while the incorporation of molecules to enhance charge or hydrophilicity is a common approach for improvement of separation capabilities and fouling reduction [132]. The use of charged molecules within the membrane matrix will generally lead to superior removal of charged molecules such as ibuprofen and sulfamethoxazole but poor removal of molecules such as carbamazepine [133]. Graphene-containing ceramic composite tubular membranes were prepared by dip-coating of a graphene-TiO_2_ mixture onto tubular Al_2_O_3_ membranes, followed by calcination. The removal of three pharmaceuticals (cephalexin (CLX), sulfamethoxazole (SMX) and caffeine (CAF)) was investigated under simultaneous electrocoagulation/electrofiltration. CLX and SMX have p*K*a values of 4.5 and 6.2, and zeta potentials of −6.14 mV and −14.45 mV respectively; this resulted in their negative charge in aqueous solution leading to rejection by electrostatic repulsion from the negatively charged graphene containing membrane, with rejections of 23% and 46% respectively under standard crossflow conditions in model water, with significantly higher removal achieved in real WWTP influent; significant improvement in removal from model water was seen using EC/EF technique. Removal from WWTP influent was almost complete without EC/EF in any case due to adsorption of pharmaceuticals onto NOM, however permeate flux was improved with increase in applied electric field strength. The p*K*a of CAF is 10.4 with zeta potential of −0.77 mV CAF, and as such very poor removal was seen.

Integrated systems for PPCP removal have been explored extensively in the literature, including the use of NF with advanced oxidation processes (AOPs) such as ozone, peroxide, UV, photo-Fenton, photocatalysis, electrocoagulation [134] and electrochemical AOPs [135], and have in many cases shown enhanced performance of NF in terms of enhanced flux and removal of organic contaminants [136]. Ozonation for example can be efficient in the removal of polar compounds such as diclofenac and carbamazepine, although not effective on clofibric acid or bezafibrate [77]. While granular activated carbon (GAC) operations alone are not particularly useful for removal of hydrophilic, polar/charged high MW compounds (including clofibric acid, diclofenac and carbamazepine), the inclusion of an NF step prior to GAC removes the majority of NOM that is present for competitive adsorption, leading to enhanced removal of PPCPs by GAC [101]. The adsorption capacity of GO and rGO has also shown promise in the removal of PPCPs, with membranes prepared for adsorptive removal of cations, anions and BPA developed with selective adsorption characteristics on UF membranes [137]. A photocatalytic cubic Ag/AgBr/GO nanocomposite showed complete degradation of diclofenac within 6 minutes under visible light irradiation [138]—integration of this type of composite within a membrane would be of great interest.

Nanofiltration can be suitable for removal of virtually all pharmaceuticals and trace organic compounds. However, a single nanofiltration membrane will not provide a complete solution to all potential PPCP contamination. For example, removal of a wide range of PPCPs from real surface waters was found to range from 0% to over 90% [87], with poor rejection often being associated with looser NF membranes and uncharged low MW molecules [73], while studies testing novel membranes incorporating surface charge modifying molecules, unsurprisingly, show good removal of molecules like ibuprofen but poor removal of molecules such as carbamazepine [133]. However, the removal of PPCPs by NF is generally above that which is possible with conventional treatment [139]. The solute characteristics and the membrane characteristics must be carefully matched, and operating parameters such as pH, ionic strength and crossflow velocity must be appropriate. It should be possible to develop an integrated system, for example in which NF membranes with different characteristics are applied in series, to provide a complete solution to PPCP contamination in drinking water treatment. However there are two issues with this—the energy costs associated with extensive NF, and the subsequent treatment of NF concentrate. The components of NF concentrate from drinking water treatment have typically been subjected previously to a range of operations from WWTP processes, or adsorption/advection during migration to the drinking water source. As such they are generally difficult to treat or biodegrade, and appropriate treatment processes for the concentrate must also be developed, such as UV/ozone treatment [140]. However, although subsequent treatment of concentrate is required, there can be savings in overall materials or energy costs, by application of advanced processes such as solar photo-Fenton or UV/O_3_ to the smaller volume concentrate rather than to the full volumetric flow of water [140,141], and overall an integrated system is recommended [142].

While much of the research outlined here is at experimental stage and has not been brought forward to commercialization, there are some developments that have brought the utilization of graphene derived membranes into the public domain for water treatment. Perforene™, for which the patent was acquired by Lockheed-Martin in 2013 [143], consists of a graphene membrane perforated with holes less than 1 nm thick, with up to 5 times improvement in membrane flux expected over existing desalination processes, as well as 10%–20% reduction in energy consumption due to pressure reduction, and fouling reduction of up to 80% with corresponding energy savings and increased membrane life [144]. They also intend to apply this system to treatment of water in the oil and gas industry rather than for desalination, where the requirement would be for nanoholes of only 50–100 nm in diameter. A start-up company (American Water Recycling) was established in 2013 out of research by the University of Texas in El Paso [145] to develop graphene-based membranes for grease removal from water [146]. Both ventures are, however, still at an early stage.

## 6. Conclusions

The incorporation of graphene based nanosheets into membranes shows great promise in enhancement of flux (for example water flux in desalination or water purification). The extremely high capacity for water transport, high hydrophilicity and tunable gap spacing of GO sheets coupled with their ease and low cost of manufacture indicates that their incorporation into a widening variety of membrane types will continue to increase. Graphene based nanosheets have been incorporated into polymeric membranes for liquid phase separation by mixing of GO/rGO with polymer followed by membrane fabrication using standard methods such as phase separation or thin film composite preparation. Graphene/GO has also been immobilized onto ceramic membranes by vacuum filtration, spin casting or drop coating, and incorporated into electrospun polymeric membranes. The enhanced hydrophilicity and decrease in surface roughness typically improves the flux and can enhance the separation efficiency due to increase in membrane charge for electrostatic exclusion. GO/rGO can enhance photocatalytic activity when incorporated into nanocomposite membranes with photocatalyic molecules such as TiO_2_, and nanofiller molecules can also act to tune the spacing between nanosheets allowing for manipulation of selectivity.

Graphene/GO/rGO functionalized membranes have exhibited superiority over conventional membrane processes in a wide range of applications, and the number of research papers concerning graphene functionalized membranes has increased exponentially in the last few years. The as-yet unrealized potential for commercialization and wide scale use of these types of membranes has great promise with many applications yet to be discovered.
